# Assessment of the quality of malaria surveillance and laboratory services for diagnosis in three districts of Gujarat state, India

**DOI:** 10.3389/fpubh.2024.1465228

**Published:** 2024-11-15

**Authors:** Jaspreet Kaur, Rajendra Baharia, Mamta Dattani

**Affiliations:** ^1^Department of Vector Genomics, ICMR-National Institute of Malaria Research, Sector-8, New Delhi, India; ^2^ICMR-National Institute of Malaria Research, Field Unit, Nadiad, Gujarat, India; ^3^Vector Borne Diseases Control Program, Government of Gujarat, Gandhinagar, Gujarat, India

**Keywords:** malaria surveillance, active case detection, passive surveillance, annual blood examination rate (ABER), assessment of laboratory services

## Abstract

**Objectives:**

Surveillance is the backbone for the control of malaria and its elimination. In the state of Gujarat, situated in the western region of India, some of the districts reported a high annual blood examination rate (ABER) for malaria. Therefore, a study was conducted to identify the underlying reasons for the increase in the ABER for malaria.

**Methods:**

Planned investigations were carried out in three of the state districts, scrutinizing records of malaria forms and other epidemiological data collected during health worker surveillance, assessment of laboratory services, and rapid fever surveys.

**Results:**

The rate of fever ranged from 8 to 57% in the primary health centers that were surveyed. Analysis of epidemiological data revealed that malaria parasite positivity was more from passive than active surveillance. Increased ABER was accounted for by multiple factors, including blood slides collected during the mass survey and contact smears, which were included in the ABER and not mentioned separately. Blood slides prepared for the migrant population were included in the ABER, but the migrant population was not counted while calculating the ABER.

**Conclusion:**

The ABER in villages surveyed varied from 1.6 to 78%, which is mainly due to indiscriminate preparation of blood slides, i.e., without fever symptoms. Addressing the key gaps identified in data recording may aid in channeling the limited resources efficiently, thereby progressing toward malaria elimination. Adequate surveillance activities, along with systematic data recording, will enable timely, informed decision-making for the effective allocation of resources, ultimately supporting malaria elimination efforts in the state.

## 1 Introduction and rationale

The period of success in global malaria control has been followed by a plateau in further advancements. Despite being preventable and curable, malaria remains a major public health issue worldwide (1). However, India has made significant progress in reducing malaria incidence, with a consistent decline in malaria cases across the country since 2000 (2). Malaria remains unstable in certain regions of the country, with Gujarat state being one of them, contributing significantly to cases caused by *Plasmodium vivax* (3). Effective malaria case detection through robust surveillance and comprehensive treatment is vital for vector-borne disease control programs aimed at reducing the disease burden in the state. This process provides crucial evidence for policymakers to identify areas and population groups most susceptible to malaria, facilitating the monitoring of changes in disease patterns. Consequently, this enables health authorities to design appropriate interventions. Additionally, evaluating and assessing surveillance systems is vital for their continued improvement (16, 17). In India, the surveillance of malaria cases is conducted through active and passive case detection as per the operational manual of the National Center for Vector-Borne Disease Control (NCVBDC) (4). In passive detection, patients visit the healthcare provider/facility for evaluation. In contrast, active case detection involves health service providers visiting households to detect malaria cases. Fortnightly, active surveillance is essential for interrupting malaria transmission. Timely collection and investigation of blood smears are crucial strategies of the government's “early diagnosis and prompt treatment” policy.

In Gujarat state, blood smear examination/collection has increased gradually from 12.68 million in 2013 to 14.78 million in 2016 (data from the state). An annual blood examination rate (ABER) of 10% is considered adequate, but a few districts of Gujarat state have reported a high annual blood examination rate (ABER). The Joint Director of the Vector Borne Disease Control Program (VBDCP) in Gujarat requested the National Institute of Malaria Research (NIMR), a Central Government agency, to independently assess surveillance and laboratory services at primary health centers (PHCs). Hence, a study was undertaken to evaluate the quality of surveillance and laboratory services in three districts of Gujarat state, viz., Anand, Ahmedabad, and Gandhinagar districts. The study aimed to assess the performance of active and passive surveillance agencies through scrutiny of records, conducting personal discussions with staff, carrying out field visits, and assessing the quality of laboratory services to patients in the study area.

## 2 Methodology

### 2.1 Study area

The study was carried out in Gujarat state, situated in the western region of India, which shares its international border with Pakistan in the northwest and national borders with the states of Rajasthan, Madhya Pradesh, and Maharashtra.

The state's three-tier public healthcare delivery system is structured into primary, secondary, and tertiary levels. The primary healthcare system is further subdivided into three categories: (i) sub-centers (SCs)/sub-health centers (SHCs) at the base level, catering to a population of 3,000–5,000; (ii) primary health centers (PHCs) serving a population of 20,000–30,000; and (iii) community health centers (CHCs) catering to a population of 80,000–1,20,000.

**Figure 1:**
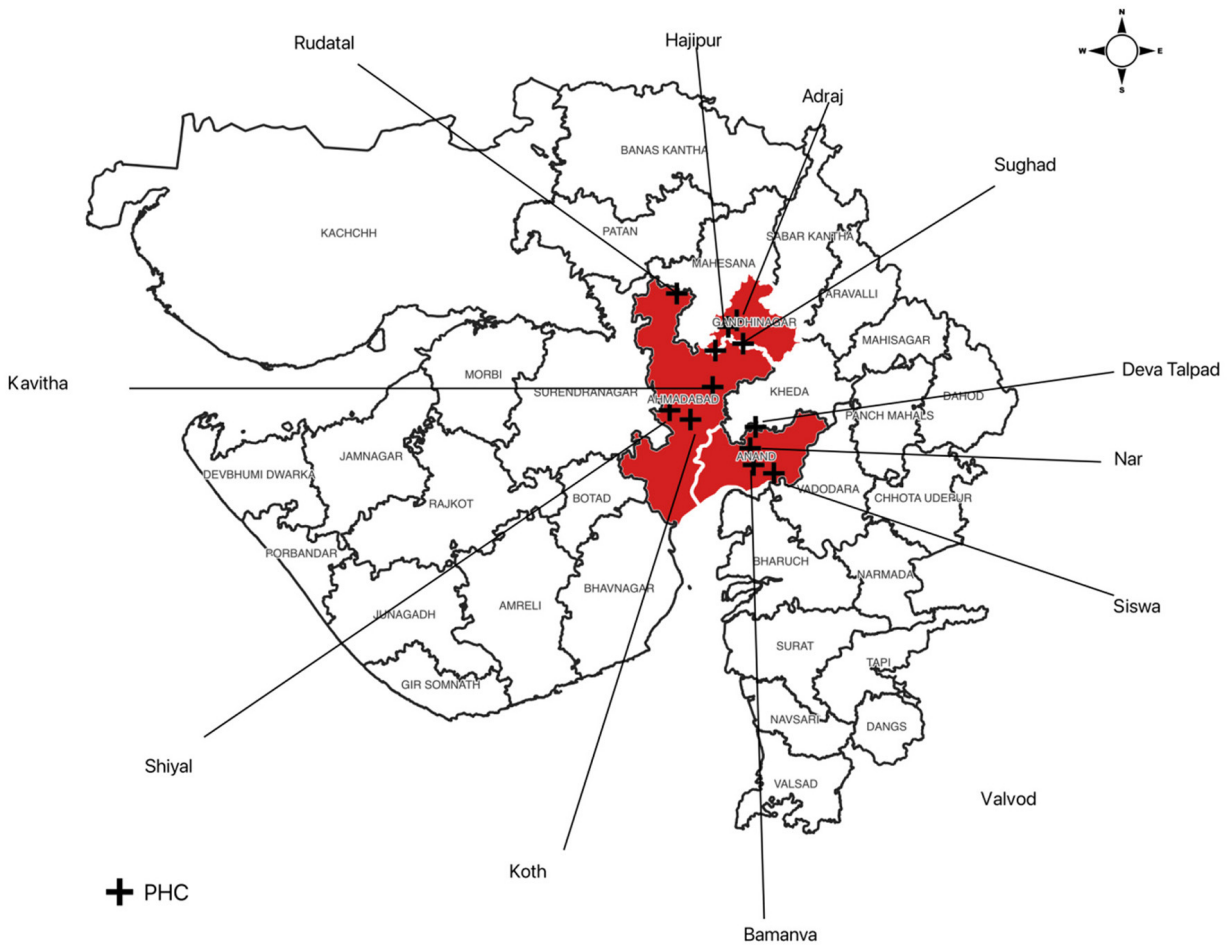
Map of Gujarat state showing the study area of 12 primary health centers (PHCs) in three districts, + representing the location of PHCs.

According to the State Programme Office, seven districts, viz., Anand, Ahmedabad, Gandhinagar, Surendranagar, Patan, Banaskantha, and Dahod, have reported >25% ABER in 2016. The ICMR-NIMR, Nadiad team, planned an investigation in three districts (Anand, Ahmedabad, and Gandhinagar). A team consisting of scientists, a technical officer, a laboratory technician, and a field and laboratory assistant visited four PHCs in each of the three districts in 2017. The study area is shown in Figure 1.

### 2.2 Selection of PHCs and villages

Based on the annual blood examination rate (ABER) in 2016, four PHCs each in three districts, namely, Ahmedabad, Anand, and Gandhinagar, showing both high and low ABERs were included in the study (Table 1). The teams conducted the rapid fever survey and visited PHCs with low and high ABERs in each district. The rapid fever survey was carried out in one village of each PHC to assess the quality of domiciliary visits of the concerned multipurpose health worker.

**Table 1:** Data on population and annual blood examination rate (ABER) of primary health centers (PHCs) and villages included in the study.

**Sub-division (Taluka)**	**PHC**	**Village**	**Population**	**ABER**
**Anand**				
Khambhat	Bamanva	Vadala	2,844	4.68
Borsad	Siswa	Valvod	7,931	41.9
Petlad	Nar	Manaj	1,859	50.67
Sojitra	Deva Talpad	Bhadkad	4,503	23.07
**Gandhinagar**				
Gandhinagar	Sughad	Sughad	2,033	78.36
Gandhinagar	Adraj	Sardhav	8,823	14.6
Kalol	Hajipur	Bhimasan	1,374	40.25
Kalol	Rancharada	Rancharada	3,226	37.35
**Ahmedabad**				
Bavala	Shiyal	Bagodara	6,292	26.3
Bavala	Kavitha	Saljada	1,953	34.1
Detroj	Rudatal	Rudatal	3,505	48.5
Dholka	Koth	Ganpatipura	7,258	1.64

### 2.3 Discussion with officers and staff

The team discussed the surveillance plan, activities, and data with medical officers, laboratory technicians, supervisors, multipurpose health workers (MPHW-M), and Accredited Social Health Activists (ASHA) of the concerned PHCs. Apart from this, malaria forms, M-1, M-2, M-3, and M-4, as well as records of MPHW (M) and ASHA, were also scrutinized to determine the sources of blood smear collection, examination, and recording/reporting.

### 2.4 Assessment of laboratory services

The malaria microscopy laboratory at each primary health center (PHC) was inspected, focusing on the available equipment, logistics, methodology, records, and cross-checked reports of blood slides.

### 2.5 Malaria surveillance in villages

According to the NVBDCP manual, active case detection (ACD) should be carried out by assigning a multipurpose health worker male [MPHW (M)] to each PHC, following the advanced tour program of the worker (4). The fortnightly domiciliary visits using “reporting formats for malaria” are the technical requirement of malaria disease management. Active case detection ensures uninterrupted linkage between the communities and the peripheral health delivery system. The main components of the door-to-door active surveillance are as follows: (a) searching for individuals with fever or those who had a fever since the last visit by the MPW, (b) collection and examination of blood smears from such cases, and (c) administration of appropriate anti-malarial(s) as per the national malaria drug policy.

Furthermore, all individuals with fever attending the PHC should be screened for malaria, and blood smears collected by MPHW (F), ASHAs, Anganwadi workers, and other agencies should be included in passive case detection. The malaria forms and records of the health facility were examined, and discussions were held regarding the schedule and methods of activities carried out by supervisors, MPHW (M), and ASHAs. The treatment of confirmed malaria cases provided by staff was also scrutinized using radical treatment cards.

### 2.6 Rapid fever survey

The NIMR teams along with local health staff (MPHW) of the village and ASHAs carried out a rapid fever survey in one village of each PHC visited during the investigation, covering a minimum of 300–500 population. A blood smear of each febrile person was prepared. The blood smear was processed, stained, and examined at the NIMR laboratory.

### 2.7 Data collection and analysis

Retrospective data on malaria surveillance, diagnosis, and treatment were obtained from each PHC and sent to Medical Officers in a prescribed format before the PHC visit.

## 3 Results

### 3.1 Surveillance and record examination

**Figure 2:**
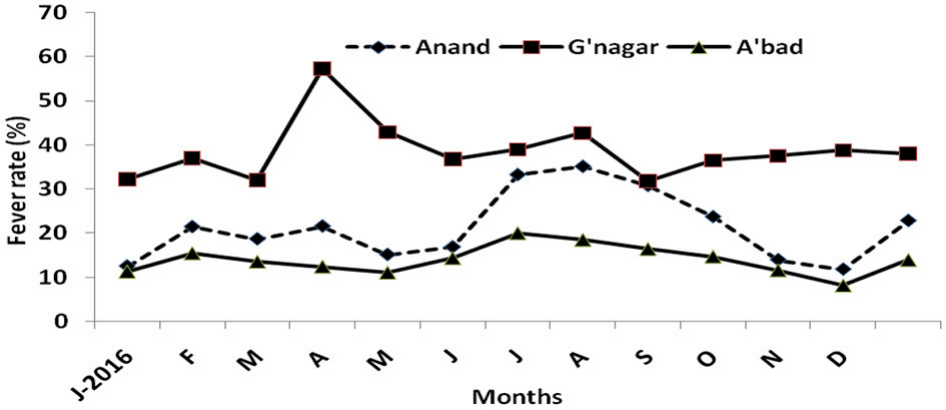
Fever rate in Anand, Gandhinagar, and Ahmedabad, based on four primary health centers in each district.

Data on monthly OPD attendance and febrile patients in four PHCs in each of Anand, Gandhinagar, and Ahmedabad districts were obtained to determine the fever rate in each district. It has been observed that the Gandhinagar district reported the highest fever rate/febrile patients (31.7–57.26%) throughout the year, followed by Anand (11.83–35.2%) and Ahmedabad (8.1–20.03%; Figure 2).

**Figure 3:**
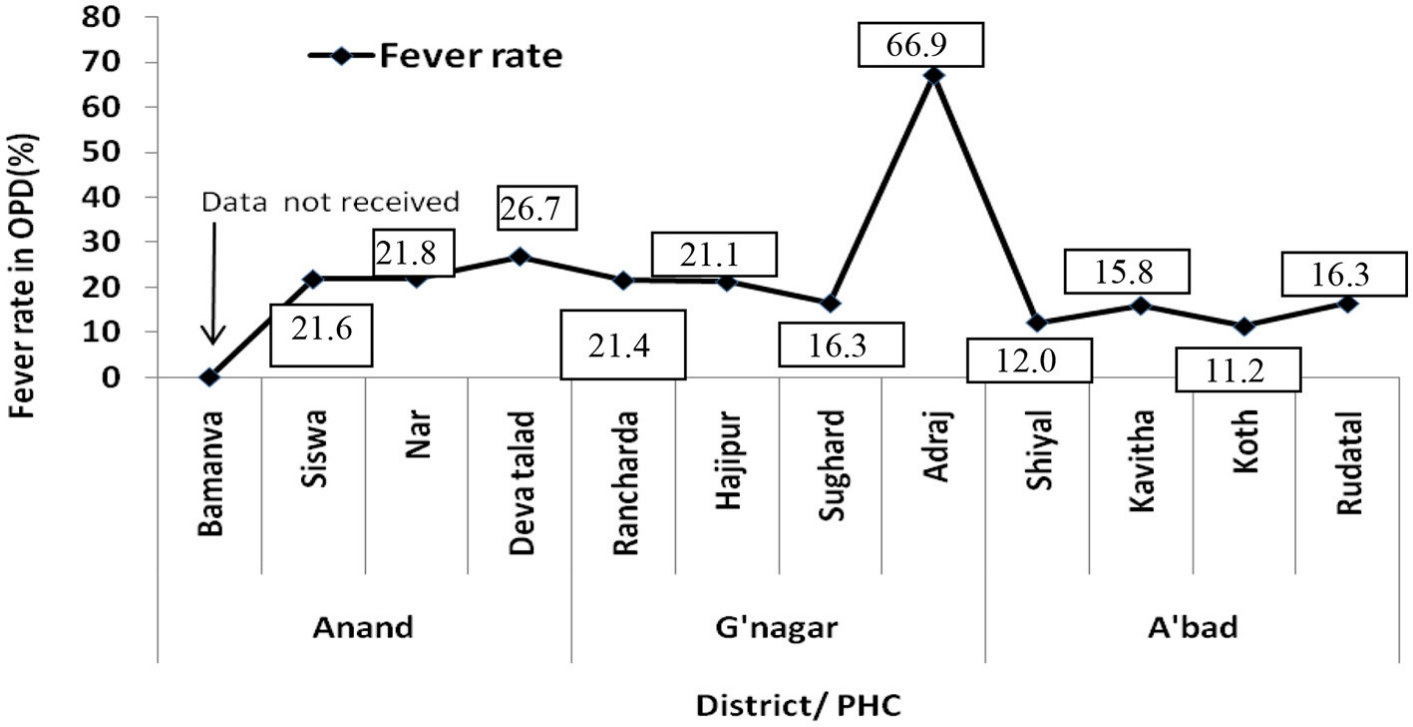
Febrile patients in OPD of different PHCs.

The PHC-wise analysis of OPD revealed > 21% of febrile patients in Anand and 16–21% in Gandhinagar except in Adraj PHC where 66.9% of OPD patients were febrile in 2016. In Ahmedabad, all four PHCs reported < 17% of febrile patients in OPD (Figure 3).

### 3.2 Agency wise blood slide collection/examination

**Figure 4:**
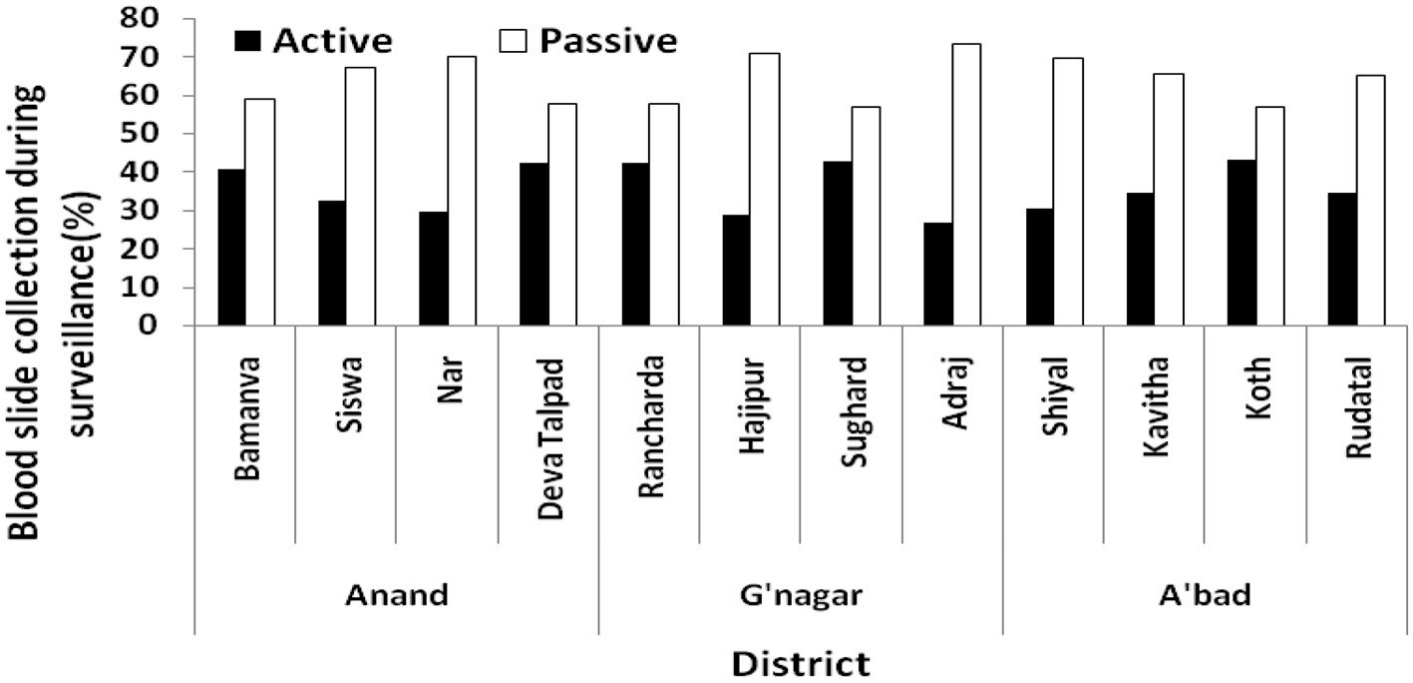
Blood slide collection by active and passive surveillance in visited PHCs.

Data on blood slide collection/examination (BSC/E) were obtained from each PHC visited in the study districts. In general, a large proportion of blood slides were collected by the passive agencies (ASHA, MPHW (F), and PHC OPD (Figure 4). The passive collection among 12 PHCs in three districts ranged from 56 to 73%. In Anand, active collection ranged from 29 to 42% among four PHCs, 26–42% in Gandhinagar, and 30–43% in Ahmedabad. In all the PHCs, the passive collection was more than the active collection, ranging from 56 to 82.5%.

Table 2 reveals that active collection in each PHC was nearly 40% of the total BSE but the positivity was very low, indicating poor quality of surveillance. In Gandhinagar, not even a single malaria case could be detected from the active collection in four PHCs (Table 2).

**Table 2:** Blood slide collection and cases diagnosed by surveillance agencies.

**District**	**PHC**	**Surveillance agency**	**BSE**	**Cases**	***P. falciparum***
Anand	4	Active	15,843	16	1
		Passive	33,281	189	13
Gandhinagar	4	Active	17,187	0	0
		Passive	28,819	31	4
Ahmedabad	4	Active	17,861	3	0
		Passive	33,012	45	1

### 3.3 Annual blood examination rate

Table 3 shows the ABERs and SPRs of the study districts over a period of time, whereas Table 4 shows the ABERs of the PHCs visited; The ABERs for PHCs visited are given in Table 3, it has been seen that the ABERs in 12 PHCs of three districts were ≥25% except in Bamanva (12.30%) PHC of Anand and Shiyal (19.91%) PHC of Ahmedabad district.

**Table 3:** Annual blood examination rate (ABER) and slide positivity rate (SPR) of study districts (12).

	**2017**		**2018**		**2019**		**2020**		**2021**		**2022**		**2023**	
	**ABER**	**SPR**	**ABER**	**SPR**	**ABER**	**SPR**	**ABER**	**SPR**	**ABER**	**SPR**	**ABER**	**SPR**	**ABER**	**SPR**
Anand	24.91	0.07	22.85	0.03	24.02	0.01	18.34	0.00	19.79	0.00	20.70	0.00	25.0	0.0
Gandhinagar	26.59	0.07	24.62	0.06	27.14	0.04	24.02	0.02	25.52	0.02	26.22	0.02	27.9	0.0
Ahmedabad	27.03	0.05	24.70	0.03	25.08	0.01	21.66	0.01	20.99	0.00	23.5	0.0	30.8	0.0

**Table 4:** Monthly (annual) blood examination rate in PHCs.

**District**	**PHC**	**J-2016**	**F**	**M**	**A**	**M**	**J**	**J**	**A**	**S**	**O**	**N**	**D**	**ABER**
		**Monthly blood examination rate**												
Anand	Bamanva	0.8	0.8	0.8	0.8	0.9	1.0	1.1	1.8	1.8	1.1	0.8	0.6	12.30
	Siswa	1.6	1.8	1.9	2.0	2.2	2.7	2.7	4.7	7.7	2.8	2.0	1.3	33.33
	Nar	1.8	2.5	3.3	2.5	2.4	3.8	5.0	5.2	4.3	2.9	2.4	2.2	29.68
	Deva Talpad	0.9	1.7	1.5	1.5	2.2	2.3	2.5	3.0	4.3	2.0	1.5	1.4	24.87
Gandhinagar	Rancharda	1.7	2.2	2.7	2.1	2.2	1.6	2.7	3.1	2.8	2.0	2.2	2.1	27.43
	Hajipur	2.2	2.9	2.4	2.7	2.2	2.9	3.9	3.4	3.6	2.7	2.7	2.7	34.27
	Sughad	1.7	2.1	2.4	2.2	2.6	3.2	3.5	2.8	2.4	2.3	2.7	2.5	30.49
	Adraj	Data not available												26.37
Ahmedabad	Shiyal	1.1	1.6	1.5	1.5	1.7	1.7	1.9	1.9	1.8	2.0	1.8	1.4	19.91
	Kavitha	1.7	1.9	2.1	1.7	1.8	1.8	2.1	1.9	2.5	2.2	1.5	1.9	31.23
	Koth	1.7	1.9	2.1	1.7	1.8	1.8	2.1	1.9	2.5	2.2	1.5	1.9	43.05
	Rudatal	1.8	2.2	2.6	2.0	2.5	2.6	3.3	3.4	3.8	3.1	1.8	2.0	31.19

The ABERs in most of the villages in the same PHC varied to a very high level and remained higher throughout the year. In all three districts, the village-to-village variation in ABERs was quite high.

### 3.4 Scrutiny of records and factors for high ABER

During discussions with laboratory technicians, multipurpose health workers (MPHWs), and multipurpose health supervisors (MPHSs) from different PHCs, along with a review of records, it was observed that the blood smears collected during various focal and mass surveys under health programs have been incorrectly incorporated into active surveillance. This practice dilutes the importance of surveillance. Areas such as brick kilns, construction sites, and new projects, if included in routine surveillance, should have their populations accounted for separately when calculating the ABER. The objectives of these focal surveys differ; hence, the data must be recorded and analyzed separately to improve control strategies in particular areas and to identify the high-risk foci. Another reason for high ABER is when confirmed malaria cases were followed by PHC staff for 28 days to assess the drug efficacy. Blood slides were prepared on day 3 and weekly during the patient's follow-up. Thus, five additional blood smears of the same patient were prepared. The workers assigned fresh serial numbers to each follow-up slide, enhancing the ABER. However, several factors contributed to a high ABER, including indiscriminate blood smear preparation during antenatal check-ups, which were subsequently added to the active or passive collections. Furthermore, high variation in the ABER has been observed due to indiscriminate blood slide preparation and discrepancies in data recording by local health workers.

### 3.5 Status of laboratory services in PHCs

The NIMR team visited 12 laboratories in the three districts to assess the services provided to patients attending the PHCs, the logistics, and the conditions of the equipment related to malaria microscopy. During surveys, it was noticed that each laboratory had separate space, adequate furniture, and availability of antimalarials. The microscope of all primary health centers was found to be in working condition except in one PHC where servicing and replacement of the lenses of the microscope were required. The team suggested that a standard operating procedure (SOP) should be provided to the Laboratory Technician to maintain the microscope. Cross-checking of blood smears collected from different PHCs was performed. A few negative (119) and positive (23) slides examined by laboratory technicians of various PHCs were taken and cross-checked at the NIMR laboratory, and no discrepancy was observed. During a rapid fever survey by the team in one village of each study PHC, 82 blood smears were collected, processed, and examined at the NIMR Lab. None of them was found positive for the malaria parasite. The results have been communicated to the medical officer of the concerned PHC. A final report was prepared, and the recommendations made were transmitted to the state Health authority.

## 4 Discussion

Malaria parasite load in the community is governed by multiple parameters, such as individual immunity (5), the presence of infected individuals, the local vector population, and environmental conditions (13). The detection and treatment of malaria cases are important requisites for the elimination of malaria (6). Early identification of cases and their radical treatment aid in reducing human reservoirs and infective vectors, thus reducing malaria transmission in the community (7). Therefore, timely collection and examination of blood smears are key elements in the malaria control strategy.

Primary health centers are responsible for various programs and additional services rendered by field staff. Enhancing focused laboratory services and ensuring staff are adequately trained will enhance the performance of PHCs (15). Therefore, it is recommended that state health authorities prioritize filling all vacant positions. The involvement of the Medical Officer of the primary health center in vector control is important for arranging timely and complete surveillance in the villages. The fortnight advance tour program of the supervisor, MPHW (M), ASHAs, and other workers should be prepared under the guidance of MO to cover the entire area in time. The fortnightly door-to-door visits are a technical requirement of malaria disease management. The domiciliary active surveillance should be conducted by MPHW (M) or ASHA by visiting each family at their homes. The blood smear of only febrile patients/persons with a history of fever within the last fortnight must be prepared, and complete details, including symptoms, should be recorded in the case recording form. Furthermore, the blood slides collected by MPHW (F), ASHAs, and Anganwadi workers or during any unscheduled visit are considered a passive collection. All PHC staff should follow the standard operative procedure for surveillance. Assessment of ASHA in a tribal setting has revealed that training needs to be provided to ASHA workers for malaria surveillance and treatment (8).

The present study elucidated reasons for the high ABER reported in some districts, which is mainly due to the issues with data recording practices. There are many brick kilns, construction sites, and developmental projects in several villages where many migrant laborers camped in temporary shelters. Such construction sites and developmental projects provide ideal conditions for vectors to breed and malaria to spread (9). Usually, slides are prepared from migrants in these areas, but their population is not considered while calculating the ABER, thereby potentially augmenting the ABER. Furthermore, during follow-up of malaria cases, PHC workers prepare additional blood smears, labeling each slide with continuous serial numbers. Whereas, the follow-up slides of an individual should bear the original registration number along-with a descendant such as A, B, C (example: if original serial number is 101, then follow-up slides number may be 101-A, 101-B). To achieve this activity's objective, the results of the follow-up slides should have been analyzed separately to determine the status of drug efficacy in the community. In addition, when blood smears prepared during unscheduled surveillance activities by ASHAs, ANMs, and other agencies are included in active surveillance, it leads to an increase in the BSC and the ABER at PHCs.

In general, the laboratory services at each PHC were found to be satisfactory. However, there is scope for improvement by providing staining coupling jars with lids, microscope servicing, and replacement of damaged lenses for laboratory technicians. Excellent logistics support will also enhance the efficacy of laboratory technicians and reduce the time lag in reporting. Availability of antimalarials (2.5 mg PQ and ACT) and RDT at PHC should be monitored, and arrangements should be made to fulfill the requirement. A brief standard operating procedure for malaria laboratory maintenance should be developed and distributed to laboratory technicians for good laboratory practices.

Studies have highlighted that adequate surveillance activities performed over some time along with proper data recording (10, 14) can impact the malaria burden in the area. Furthermore, it has also been suggested that surveillance activities indicated by ABER can have an impact on other metrics used in malaria, such as annual parasite incidence (API) (11), thereby highlighting the importance of ABER and surveillance activities. The present study has a few limitations. The study was conducted at a limited number of sites primarily due to the restricted availability of data from different sites and time constraints. Another limitation is that recent data from various PHCs could not be analyzed and included in the study. In spite of these limitations, the study has provided essential insights into the factors responsible for augmented ABER in various PHCs of different districts of Gujarat state. We have identified vital gaps that, when addressed, will help build an effective surveillance system, thereby facilitating malaria elimination in the state.

## 5 Conclusion

The analysis of OPD attendance and febrile patients showed that fever rates ranged between 16 and 26% in different primary health centers except one. However, the indiscriminate blood smear preparation in each PHC has exaggerated the ABERs in the studied districts. The study analyzed the factors exaggerating the ABERs in different districts of Gujarat state and identified the gaps that need to be addressed through state health authorities. Proper surveillance activities, along with an appropriate recording of data, will aid in monitoring the disease progression and channel the use of limited resources. This approach will help reduce malaria transmission and achieve zero indigenous cases in the state, leading toward malaria elimination in the near future.

## Data Availability

The original contributions presented in the study are included in the article/supplementary material, further inquiries can be directed to the corresponding author.
